# Evidence from ERP and Eye Movements as Markers of Language Dysfunction in Dyslexia

**DOI:** 10.3390/brainsci12010073

**Published:** 2022-01-01

**Authors:** Aikaterini Premeti, Maria Pia Bucci, Frédéric Isel

**Affiliations:** UMR 7114 MoDyCo, CNRS-Université Paris Nanterre, 92001 Nanterre, France; mariapia.bucci@gmail.com (M.P.B.); fisel@parisnanterre.fr (F.I.)

**Keywords:** dyslexia, reading, attention, eye movements, ERP

## Abstract

Developmental dyslexia is a complex reading disorder involving genetic and environmental factors. After more than a century of research, its etiology remains debated. Two hypotheses are often put forward by scholars to account for the causes of dyslexia. The most common one, the linguistic hypothesis, postulates that dyslexia is due to poor phonological awareness. The alternative hypothesis considers that dyslexia is caused by visual-attentional deficits and abnormal eye movement patterns. This article reviews a series of selected event-related brain potential (ERP) and eye movement studies on the reading ability of dyslexic individuals to provide an informed state of knowledge on the etiology of dyslexia. Our purpose is to show that the two abovementioned hypotheses are not necessarily mutually exclusive, and that dyslexia should rather be considered as a multifactorial deficit.

## 1. Introduction

Reading is a complex skill that entails oculomotor and cognitive processes such as eye movements (convergence, saccades and fixations), visual perception and various language processes in charge of letter, lexical, syntactic, and semantic analyses [1]. Reading depends on the acquisition of phonological awareness, which is the knowledge that language is composed of syllables and phonemes and the ability to associate distinctive visual signs (graphemes) to their corresponding units of speech sound (phonemes) [2]. A deficiency in phonological awareness is considered to be a prevalent cause of dyslexia [3,4].

Dyslexia is a specific and significant impairment in the development of reading skills, despite normal intelligence, adequate schooling and no neurological or sensory problems [5]. Dyslexia is found in different languages independently of language orthographic transparency [6] (i.e., the consistency with which orthographic letter sequences and orthographic phoneme sequences are mapped in both directions, from reading to spelling and vice versa; orthographic transparency thus distinguishes two groups of languages: transparent, or languages with swallow orthography, and non-transparent, i.e., languages with deep orthography). This language-specific disorder is characterized by difficulties in recognizing words, deficits in reading speed and fluidity, spelling difficulties and problems with reading comprehension [7].

After more than a century of research, the etiology of dyslexia still remains debated [8]. The most prevalent theory of developmental dyslexia nowadays is the phonological deficit theory [9], which posits that dyslexic readers present poor phonological awareness, poor verbal short-term memory and slow lexical retrieval [4]. However, it is disputable whether the phonologic representations of dyslexic individuals are unspecified or whether it is the access to these representations that is impaired, maybe due to the difficulty in separating the two hypotheses (quality of representations vs. access to the representations) [10,11,12].

On the other hand, many researchers consider that the phonological deficit hypothesis is not the only nor the most relevant way to explain dyslexia. Some studies have reported a reduced visual attention span in dyslexics that may result in a limitation in the number of letters processed in parallel. This disorder, known as visuo-attentional deficit, can lead to abnormal eye movements during reading in dyslexic individuals [13,14,15].

These two fundamental hypotheses of dyslexia have been extensively investigated by two scientific communities, each of which uses different techniques to detect and analyze dyslexic individuals’ characteristics. Mental chronometry (reaction times) and electroencephalography of event-related potentials (ERP) are used to test the phonological deficit hypothesis by examining the time course of the phonological process during reading, whereas eye movement recordings are used to examine the visuo-attentional deficit hypothesis.

In this article, we review current research on both event-related brain potentials (ERP) as well as eye movement recordings in the dyslexic population in order to propose future research directions that could embrace these two hypotheses. In our view, the simultaneous recording of ERP and eye movements in dyslexic individuals may improve our knowledge of the multidimensional causes of this language disorder.

The purpose of this paper is to override frontiers between the two scientific communities and encourage scholars to interact each other.

In the first part of this article, we review selected ERP studies used to examine brain activity in dyslexic and control individuals. We then review the results on eye movement studies conducted in these two populations.

## 2. ERP Recordings in the Dyslexic Population

Researchers have used several techniques to study brain activity in readers with developmental dyslexia. Among them, electroencephalography of event-related brain potentials (ERP), due to its high temporal resolution, enables the disturbed language processes involved in reading to be studied. ERP varies on three dimensions: polarity (positive, negative), peak latency (i.e., the time between the stimulus onset and the maximum), and topography of the surface. Critically, language processing at different levels of linguistic analysis (phonology, prosody, morphology, morphosyntax, syntax, semantics, pragmatics, and lexicon) is thought to be, respectively, reflected by a specific ERP (for a review, see [16]). In several studies, quantitative differences have been found with reduced amplitudes and/or increased latencies of the ERP in dyslexic participants compared with matched control ones.

This part of the review concerns ERPs focusing on word recognition during reading tasks. Consequently, we decided to focus on the components thought to reflect the different stages of processing from letter to word in dyslexic and non-dyslexic participants, i.e., N170, N320, N400 and LPC (see also Table 1).

### 2.1. N170

Apart from auditory perception, dyslexia is mostly a language-specific disorder that is usually detected by a reading speed deficit. The initial phases of reading, i.e., within the first 200 ms of stimulus presentation, are related to specific visual brain processes. These processes occur in inferior occipito-temporal areas [32], and more precisely, in an area located in the mid-portion of the left fusiform gyrus, which is assumed to support visual word form processing as it is activated in response to word-like stimuli [33]. This area, known as the visual word form area (VWFA), is linked to the N170 component in studies combining fMRI and ERPs. The N170 component (also called N1 [17] in the literature) is a negative waveform that peaks at around 150–220 ms after stimulus presentation onset in the left occipito-temporal region at the surface of the scalp. It has been generally associated with orthographic processing, since its amplitude is larger for visual alphabetic stimuli of a target language (words, pseudowords, non-words) than for visual non-alphabetic stimuli (for instance, symbol strings) [34]. It is assumed that the N170 may be related to the prelexical stage of word recognition [35]. Many studies have shown that the initial stage of reading, i.e., visual orthographic processing, is deficient in dyslexia.

However, the literature fails to show consistent evidence as some studies report a difference of N170 effect (difference between alphabetic and non-alphabetic conditions) between dyslexic participants and controls [22,23], while other studies did not find such a difference in either children [17,19], preadolescents [18] or adults [20,21]. We first present studies which found a difference of N170 effect between dyslexic and control individuals.

Maurer et al. [17] tested German children from kindergarten to second grade with and without dyslexia (or at a high risk of dyslexia, in the case of kindergarten children) using a detection task consisting in detecting whenever each string of letters or symbols was or was not repeated. An N170 effect was only observed in the group without dyslexia. Critically, in dyslexic children in second grade, the amplitude of the N170 did not differ between words and symbol strings. This result suggests that dyslexic children have a lower sensitivity to letter strings than controls.

Similar results (no N170 effect for dyslexics) were found in Portuguese preadolescent dyslexics in a letter/symbol decision task [18] (during this task words, word-like stimuli and symbol sequences were presented one at a time to the participants who had afterwards to decide whether a letter/symbol was included in the previously presented stimulus), and in German children by Hasko et al. [19] using a phonological lexical decision task (i.e., participants had to decide whether a visually presented stimulus sounded like a real word or not).

In the same vein, a lack of N170 effect between alphabetic and non-alphabetic stimuli was also reported in dyslexic adults [20,21]. For example, Mahé et al. [20], using a lexical decision task, failed to show an N170 effect in French dyslexics. In contrast, the results revealed an N170 effect in non-dyslexic participants.

Shany and Breznitz [21] conducted a study focusing on Hebrew-speaking dyslexic adults. The authors split participants into three subgroups based on performance in word reading (accuracy disabled, rate disabled and a group that presented both accuracy and rate impairments). They used a letter detection task in which participants were presented with two different letters (one target letter and one non-target letter) in Hebrew and had to detect when the target letter was presented on the screen. They compared N170 amplitudes between the three groups and found that the N170 amplitude was smaller for the accuracy impaired group but similar for the rate-impaired group. Interestingly, the most reduced N170 amplitude was found for the group of dyslexics that presented a mixed profile (i.e., both rate and accuracy deficit). The data suggest the presence of diverse reading impairment profiles, which are based on selective abnormalities in reading rate vs. accuracy.

Together, all these data showing similar processing of verbal (linguistic/alphabetic) and non-verbal (non-linguistic/non-alphabetic) stimuli in dyslexics as indexed by the absence of an N170 effect suggest that dyslexics have a reduced level of print sensitivity and that they have deficits in visual orthographic processing during reading.

In contrast, other studies found an N170 effect (difference between alphabetic and non-alphabetic stimuli) in both dyslexic and non-dyslexic individuals, whether in children [22] or adults [23]). Maurer et al. [22], using a repetition detection task with words, pseudowords, symbol strings, and pictures in a longitudinal study on German children (from the second to the fifth grade), found that the N170 effect which was initially reported only in non-dyslexic children was also observed later in dyslexics of the fifth grade. In addition, another study found similar results between Portuguese dyslexic and non-dyslexic adults, i.e., an N170 effect during reading in a letter/symbol decision task (the experimental task was the same as in the study by Araújo et al. [18]).

This inconsistency in results with respect to other studies already cited may be attributed to the severity of dyslexia in the individuals participating in the studies [17]. In the study by Maurer et al., the population of fifth graders examined was 1.29 standard deviations below the normal mean reading range. In the study by Araújo et al. [23], dyslexic adults’ word reading scores were 1.5 standard deviations below the scores of control individuals, whereas in other studies [20], dyslexics presented deficits at least two standard deviations below the mean reading range.

Another explanation of the inter-studies inconsistency may be the task difficulty. It is possible that the lexical decision task used in some studies highlighted the difficulties in dyslexia and generated a greater N170 effect as opposed to a repetition detection task [22] or a letter/symbol detection task [23] that did not point out these difficulties. Finally, we have to note that, since in the study of Maurer and al. the task was exactly the same for second and fifth graders, it is possible that the task was too easy for the dyslexics in fifth grade.

### 2.2. N320

In many studies, variations in ERP components associated with phonological processing, such as the N320 component, have been found between normal and dyslexic readers. The N320 effect reflects a larger N320 response for phonologically legal stimuli (e.g., words, pseudowords) than for phonologically illegal ones (e.g., consonant strings, non-words). This effect is largely distributed over the left temporal and temporoparietal sites and peaks at around 310–350 ms [34,36]. The N320 effect was found in different tasks such as rhyme judgment [34] or silent reading tasks [36]. Some ERP studies found no N320 effect since there was not a significant difference of the N320 amplitude between phonologically legal and illegal stimuli with respect to dyslexic participants.

Araújo et al. [23], using a letter/symbol detection reading task, found no N320 effect for dyslexics since there was no significant difference between pseudowords (phonologically legal stimuli) and non-words (phonologically illegal stimuli). Reduced N320 amplitudes have also been found in French dyslexic adults during a reading aloud task [24].

However, an earlier study by the research group of Araújo et al. [18] found no significant N320 effect between pseudowords (phonologically legal stimuli) compared to consonant strings (stimuli with no phonological representation) in dyslexic preadolescents. They did, however, find more left-lateralized topographic differences in controls than in dyslexics who did not show any lateralization effect. This absence of hemispheric asymmetry in the group of dyslexics may indicate that reading abilities in dyslexics are disturbed and follow an abnormal developmental route. These hemispheric variations may indicate a lack of consolidation of the left hemisphere reading network in dyslexic preadolescents and suggest the involvement of a compensatory processing strategy in dyslexia [18].

It should be mentioned, however, that there were significant differences in experimental methodology, which could explain the different results obtained in these studies; both the age of participants (preadolescents versus adults) and the type of task used were different. It is possible that the explicit reading aloud task used in the study by Mahé et al. [24] implicitly forced participants to apply grapheme–phoneme conversion rules and therefore highlighted their phonological processing difficulty.

### 2.3. N400

As already mentioned in the introduction, impairments in the transduction of graphemic into auditory phonological codes are considered as the main cause of dyslexia. According to many ERP studies, this difficulty is reflected in the N400 component (pioneer studies by Kutas and colleagues in the neurocognitive processing of language, associated the N400 with lexical–semantic integration [37]). N400 is a negative deflection which occurs at 200–600 ms after stimulus presentation in centro-parietal electrodes during language processing. N400 is associated with lexical access, as indicated by the modulation of the N400 amplitude as a function of lexical frequency [38]. Finally, more recently, Hasko et al. [19] have proposed that N400 could also reflect the mechanism of grapheme–phoneme conversion. Further investigation should specify the exact role of this component for conversion. Given the late onset of N400 and the overall timing of word recognition, a lexical–semantic interpretation seems far more reasonable than one based on grapheme–phoneme conversion.

N400 studies on dyslexics have found mixed results. Some authors failed to find a significant N400 effect of dyslexic individuals when compared to non-dyslexic ones. For instance, Hasko et al. [19] found no N400 effect in dyslexic children, since they presented reduced N400 amplitudes for all orthographic stimuli used in the experiment (words, pseudohomophones, pseudowords). They suggested that reduced N400 amplitudes in dyslexic children imply less well-defined orthographic representations or difficulties accessing the orthographic lexicon and applying grapheme–phoneme conversion rules.

Rüsseler et al. [25] recorded the N400 component in phonological rhyme, semantic and syntactic judgment tasks in German participants (in this task, two pairs of words were presented to the participant, who had to decide whether the pair rhymed, had a semantic relation or had the correct syntactic gender). They found that, with respect to the rhyme judgment and the gender judgment tasks, N400 latencies were delayed (for the rhyme judgment task: 272 ms, and for the gender judgment task: 504 ms) in adults with dyslexia in comparison with controls (for the rhyme judgment task: 240 ms and for the gender judgment task: 440 ms). In addition, they found a longer latency of the N400 component in dyslexic individuals in the phonological judgment and in the semantic judgment tasks. However, they failed to find an N400 effect difference between the two groups. The delayed N400 amplitude on the rhyming judgment task was interpreted as reflecting phonological impairments in dyslexics. These results also suggest difficulties in other non-phonological reading processes. In particular, dyslexics seem to make greater efforts and take longer to process semantic and syntactic information. Johannes et al. [26] used a word-repetition task of frequent and infrequent English words, in which adults with and without dyslexia had to decide whether a word had been seen for the first or second time. The repetition effect, i.e., the difference between N400 amplitude in the first and second presentation, was smaller for high-frequency words than for low-frequency words. This frequency effect was larger in dyslexics than in controls.

McPherson et al. [27] used the N400 component in order to examine English adolescents with a history of reading difficulties, after being separated into subgroups based on good or poor phonetic skills in a task with rhyming and non-rhyming pictures (i.e., participants had to look at pictures and decide whether the names of two sequentially presented pictures rhymed (e.g., pear, bear) or not (e.g., tree, book)). Critically, while participants with good phonetic skills showed an N400 effect between pictures whose names rhymed with the prime, as opposed to pictures that had names that did not rhyme with preceding pictures, participants with poor phonetic skills did not. This differentiation in N400 effect between two subgroups of readers with reading difficulties and with either good or poor phonetic skills suggests the existence of specific subtypes of reading disability and evidences a decrease in neural capacity and/or activation during phonological processing in the readers with poor phonological skills [27].

However, other authors failed to show a difference in the N400 effect between individuals with dyslexia and normal readers. Bonte and Blomert [28] used a lexical decision task in dyslexic children in order to avoid the requirements associated with explicit phonological tasks, such as meta-phonological processing tasks. They found that the N400 effect of dyslexic children was similar to that of non-dyslexic children. This result may indicate that processing at a later phonological/lexical level is not disturbed in dyslexics. Consistent results were found by Silva-Pereyra et al. [29] in a word and figure categorization task. The authors failed to report differences in the N400 component between children who were poor readers and control children. This inconsistency in results may be due to many reasons, such as the task, stimulus type, participants‘ age and/or reading impairment.

### 2.4. LPC

Another component that has been associated with reading is the LPC (late positive component or late positive complex) occurring between 500–800 ms after stimulus presentation over the left centro-parietal electrodes [39,40]. The functional role of the LPC component is debated. Typically, LPC is associated with word recognition memory, since its amplitude is higher for correctly recognized words as opposed to new words in an “old/new” word recognition task [39]. It is also associated with the learning of new words and pseudowords [41,42] and with the integration of the meaning of newly learned words into semantic memory. In addition, Hasko et al. [19] surmised that LPC might reflect access to the phonological lexicon. With respect to dyslexia, reduced LPC amplitudes have been found in dyslexic adults [43], adolescents [30] and children [19,31]. For example, and as far as word recognition memory is concerned, Schulte-Körne et al. [30] used a recognition paradigm with previously presented pseudowords and symbols in ten-year-old dyslexic children; in this task, participants had to study a list of pseudowords and symbol strings and then decide whether the presented stimulus had already been seen. The authors found reduced LPC amplitudes in dyslexic children in response to pseudowords, whereas no intergroup difference was found for symbol strings. They suggested that these findings may indicate a specific memory deficit in word recognition. As far as phonological representation is concerned, Hasko et al. [19] showed that dyslexics failed to present an LPC effect between stimuli that have a phonological entry in the mental lexicon (words and pseudohomophones—i.e., stimuli that are pronounced just like a word in the target language but that are orthographically incorrect) and stimuli that do not have an entry in the mental lexicon (pseudowords). In contrast, control children showed larger amplitudes for words and pseudohomophones compared to pseudowords. These results suggest that the access to phonological representations of words may be impaired and/or that phonological representations may be underspecified [19] in dyslexic individuals. This hypothesis was later supported by Wachinger et al. [31], who used a word processing task in a longitudinal study with German children between kindergarten and second grade. In this task, children were asked to read a word or look at a picture (control condition) and to decide whether it matched an acoustically presented word. They found reduced LPC amplitudes in dyslexics only during the word and not during the picture condition. According to the authors, this result indicated that, since the access to phonological representations in the word condition takes place via grapheme–phoneme conversion (during the beginning of reading acquisition) and via semantic information in the picture condition, the difference in the LPC only in the word and not in the picture condition might be an important assumption in favor of an impaired access to the phonological representations of words [10,11,12].

Taken together, all these results concerning ERP studies suggest that dyslexics present deficits at different processing stages during written language processing, from sensitivity to characters to the retrieval of lexical information in memory. Interestingly, these difficulties in dyslexics seem to be language-independent since they are also observed in languages with different degrees of orthographic transparency.

## 3. Eye Movement Recordings in the Dyslexic Population

It is well established that eye movement recordings during text reading can provide valuable information about potential visual deficiencies in dyslexia. Eye tracking is a technique that not only provides an objective view of the reading process in real time but is also independent of any sort of verbal response since it does not assign the subject any additional task. Good control of eye movements is fundamental for reading. During reading, the central neural system has to coordinate both eyes (binocular coordination) horizontally in the text direction (to the left), vertically and/or obliquely (in order to start a new line) by a series of saccades (i.e., rapid, ballistic eye movements that direct the eyes onto the target to be fixated [44]) and of fixations (i.e., the maintenance of the visual gaze on a single location between each saccade [45]). In addition, vergence movements take place, i.e., movements of both eyes in the opposite direction in order to adjust both eyes to the distance at which the word is written to ensure correct fusion of it.

A large amount of data in studies focusing on eye movements has shown that dyslexic individuals have abnormal eye movement patterns. Over the years, almost all types of eye movements (saccades, fixation, vergence, smooth pursuit) have been studied and have shown different patterns between dyslexic and non-dyslexic subjects. This review concerns only saccades and fixations. In this review, we present some of the fundamental studies that give us a clear-cut image of the actual situation on eye movements in dyslexia (see also Table 2).

To begin with, many studies examined saccade performance in dyslexics. In particular, they examined the number and amplitude of progressive saccades (pro-saccades, from left to right) and backward saccades (saccades from right to left). Dyslexic subjects present more frequent saccades of smaller amplitude during a reading task [46,48,50,52,54,55,56,73]. In addition, many studies have mentioned a high number of backward saccades in order to re-fixate the word [50,57,58,59,73].

Apart from saccades, fixation is quite important in reading. Fixation has also been reported to be abnormal between dyslexic and non-dyslexic subjects. Dyslexics display a high number of fixations [46,48,50,54,62,63] of longer duration [46,48,50,54,63,64,65]. Other studies have also shown significant instability during fixation [58,74].

Lastly, studies examining binocular coordination in dyslexic individuals found poor binocular coordination during and after the saccades in reading and non-reading tasks [65,67] and during prolonged fixations [68,75].

Over the years, a methodological question arose as to whether linguistic or non-linguistic tasks should be used in order to test the eye movement performance of dyslexic subjects. On the one hand, in order to examine whether a visual/oculomotor deficit in dyslexia can cause atypical eye movement patterns in dyslexic individuals, many studies used non-verbal tasks in order to eliminate the side effects due to the linguistic processing implicit in reading. On the other hand, the use of non-verbal tasks in the examination of eye movements cannot be the best predictor to test differences that influence reading skills [14]. For this reason, many studies examined eye movements in dyslexics during both verbal and non-verbal tasks (see also Table 3).

Pavlidis was the first to show atypical eye movements in dyslexic participants by using a simple non-verbal task of sequentially tracking moving light sources [58]. In this task, participants were asked to follow five emitting lights (LEDs) in a horizontal array that flashed sequentially one at a time. In order to record eye movements, Pavlidis used a homemade photoelectric device that permitted him to distinguish fixations on distinctive letters of a word. Dyslexic participants [58] made approximately double the number of regressions (i.e., backward saccades) compared to normal readers, with a larger amplitude, and exhibited unstable fixation. These findings suggested a central and/or oculomotor impairment in dyslexia; Pavlidis hypothesized that the eye movement pattern of dyslexic participants could serve as a differentiating factor in dyslexia [58].

Note, however, that some studies carried out in the same period as Pavlidis’ studies failed to find abnormal eye movements in dyslexic children. Brown et al. [47] found that children with dyslexia presented similar typical saccade patterns to age-matched control children. Similarly, Stanley et al. [60] failed to replicate Pavlidis’ findings of atypical eye movements in dyslexic and control children; they only found individual differences among the dyslexics. Olson et al. [49], using almost the same experimental procedure as Pavlidis, failed to report any differences in terms of the number of saccades and of regressions and the stability of fixations between dyslexics and controls. These findings were replicated by Black et al. [51] who found no significant group differences during sequential saccade recordings (number of pro- and backward saccades).

The absence of atypical eye movements in dyslexics was also found in reading tasks. Hyona et al. [61], comparing eye movement patterns during reading aloud, found no qualitative difference in the fixation patterns of dyslexic and non-dyslexic children; the frequency and the length of words were processed in the same way by the two groups of participants.

Martos and Vila [50] tested both verbal (reading two texts of different levels of reading difficulty) and non-verbal tasks in order to examine eye movements in different kinds of subjects (dyslexic, retarded and normal readers). Dyslexic readers showed more pro saccades and backward saccades of smaller amplitude as well as longer fixations compared to normal and retarded readers, independently of text difficulty. This finding suggests that the abnormal eye movements reported in dyslexics are independent of the reading process. Some years later, Eden et al. [48] examined fixation, vergence amplitude, saccade and smooth pursuit in dyslexic children and compared these results to their phonological capacity. They found that dyslexic children present unstable fixation of small targets small vergence amplitudes which were associated with poor phonemic awareness, fixation instability at the end of saccades independently of poor phonological ability and poor smooth pursuit. They also found that dyslexics with small vergence amplitudes always had poor phonemic awareness. However, poor fixation control was found in dyslexics with or without poor phonological ability. The authors made the assumption that the atypical eye movement pattern in dyslexics in a non-reading task demonstrates that oculomotor abnormalities are not only due to language problems. It should be pointed out here that Eden et al. used a modified synoptophore in order to record children’s eye movements, i.e., a different apparatus from the one used by Pavlidis et al. [48].

This inconsistency of results concerning the eye movement patterns of dyslexic individuals is also found within the same dyslexic population between reading and non-reading tasks. Some studies have provided evidence of abnormal eye movements in dyslexic populations in reading and in non-reading tasks. For example, Jainta and Kapoula [67], who examined saccades and vergence control during text reading in dyslexic children and while freely exploring a painting for 30 s, found poor binocular coordination in both tasks among dyslexic children. Similarly, Bucci et al. [69], who examined ocular motor characteristics in French dyslexic children and in two groups of non-dyslexic children matched in chronological and reading age during text reading and during visual search, found impaired ocular motor characteristics (poor fusional vergence capabilities and poor binocular coordination) in dyslexic children and in reading age matched controls in both reading and non-reading tasks as compared to chronological age-matched non-dyslexic children. In addition, Bucci et al. [70] found poor binocular coordination of saccades in dyslexic children compared to non-dyslexic children of comparable age in both reading single words and in a task requiring fixation of a single LED. According to the authors, the abnormal eye movement patterns detected in dyslexic children “suggest a deficiency in visual attentional processing as well as an immaturity of the ocular motor saccade and vergence systems interaction”.

On the contrary, some other studies only found deviant eye movements in dyslexics during reading tasks. Kirkby et al. [71] showed that binocular coordination was task dependent. English dyslexic children showed a significant increase in fixation disparity only during the reading task, whereas they had a similar pattern of eye movements as adults and typically developing children during a non-reading task (dot scanning). Similarly, Trauzettel-Klosinski et al. [72] found atypical eye movement patterns in German dyslexics solely for reading words and not for naming pictures. De Luca et al. [46] also found a similar performance between Italian dyslexic and non-dyslexic children during a fixation task. On the contrary, dyslexic children showed more frequent and smaller rightward saccades as well as longer fixation times while reading short passages. Hatzidaki et al. [66], who examined the impact of the effects of dyslexia on various processing and cognitive components, found that Greek dyslexic children produced more and longer fixations only during text reading, whereas their eye movement pattern during a visual search task was similar to that of non-dyslexic participants. The use of a visual search task in these study was motivated by the hypothesis that if the atypical eye movement pattern of dyslexic participants during reading is due to an oculomotor deficit [58], this deficiency might also influence the performance of other non-verbal tasks that require attentional control. To sum up, some studies that compared reading and non-reading tasks found ocular motor deficits solely in tasks associated with reading and higher-level processing. Further studies examining both oculomotor performance as well as neurophysiological processing during reading in dyslexia are needed. Aside from the most famous theories according to which impaired phonological processing is the cause of dyslexia, many theories have argued that visual/oculomotor deficits might exist in dyslexics [65]. Deficits are even assumed to be related to a dysfunction of the magnocellular system (part of the visual system that allows rapid perception of movement, form, and changes in brightness). A deficit in the magnocellular system of dyslexic participants was first mentioned by Galaburda et al. [76]. According to Stein and Walsh [77], the atypical eye movement patterns of dyslexics may be due to “abnormalities of the magnocellular component of the visual system, which is specialized for processing fast temporal information”.

Other eye tracking studies focusing on dyslexic individuals have concluded that the abnormal eye movement patterns of dyslexics are not the cause but the reflection of their reading difficulties. Hyona and Olson [61], who examined the word frequency effect, found no difference between dyslexic and control individuals; fixation duration, i.e., the number of fixations and regressions in response to high- and low-frequency words, was similar to that of normal readers. Likewise, Pirozzolo and Rayner [53] found that eye movements (fixations, saccades and regressions) of dyslexic individuals were similar to those of reading age matched regular readers. Lastly, Rayner [78] found that the eye movements of dyslexic children were similar to those of non-dyslexic children when the latter were given a text that was quite difficult for them. Together, all these findings [53,61,78] showing similar eye movement patterns between dyslexics and non-dyslexics suggest that dyslexics’ processing deficit is not situated at a perceptive visual level but must be sought out at a higher linguistic level.

To the best of our knowledge, there are only two recent studies that combine both EEG and eye-tracking in dyslexia. Christoforou et al. [79] used a letter rapid automatized naming (RAN) task in Cypriot children (mean, 9.79 years). The stimuli used were pairs of letters either phonologically similar (e.g., β-θ, ε-υ; beta-theta, epsilon-upsilon) or phonologically different (e.g., β-ε, θ-υ; beta-epsilon, theta-upsilon) or visually similar (e.g., ζ-ξ, ρ-φ; zeta-xi, rho-phi) or visually different (e.g., Ζ-Ξ, Ρ-Φ). Group differences were found only for the phonological and not for the visual stimuli. Note, however, that in this study, eye trackers were used just to verify where children were fixating. The authors suggested the importance of carrying out studies on dyslexia combining both neurophysiological and eye gaze data. Jakovljevic et al. [80] studied the effect of colored overlays and backgrounds in 18 dyslexic Serbian children and 18 controls combining different oculomotor markers and neural oscillatory responses. They showed that the dyslexic children presented not only abnormal oculomotor patterns but also higher values of frequency bands (e.g., beta between 15 and 40 Hz) with purple overlay. The goal of this study was to see if there was a link between physiological factors and color changes in the text and background when reading in dyslexic and non-dyslexic children.

Although these two studies did not examine different deficits existing in dyslexia, they underlined the importance of combining different neurophysiological recordings in order to have a clear-cut image of the dyslexics’ response with both EEG and eye movement’s data.

## 4. Discussion

The goal of this article was to review current research focusing on both event-related brain potentials (ERP) as well as eye movement recordings in the dyslexic population to propose future directions in which these two methods could be tested by recording simultaneously ERP activities and eye movements in dyslexic individuals. The review was organized in two main parts, corresponding to the two main hypotheses put forward to account for dyslexia; in the first part, ERP studies, which are based on the phonological deficit theory of dyslexia (impaired grapheme–phoneme conversion rules), and in the second part, studies that examined eye movements in dyslexic individuals and that draw on the visuo-attentional deficit hypothesis (reduced visual attentional span in dyslexia) that can lead to abnormal eye movements during reading in dyslexia.

In the first part, we focused on studies that used reading related ERPs and specifically the N170, N320, N400 and LPC components. In the majority of the studies, dyslexics presented attenuated amplitudes and/or delayed latencies of the ERPs when compared to normal readers. Each of the abovementioned ERP components is associated with different processing stages in language comprehension and the diminished amplitudes and/or the delayed latencies may indicate a deficiency in these processing stages. In detail, the absence of an N170 effect (i.e., the difference between alphabetic and non-alphabetic stimuli) found in dyslexic children and adults suggests that they have a reduced level of print sensitivity. Moreover, one can assume that they have deficits in visual orthographic processing during reading.

Additionally, the absence of an N320 difference between phonologically legal and illegal stimuli in dyslexic individuals may indicate poor phonological processing in dyslexia.

The N400 component, which is associated with grapheme–phoneme conversion rules and to access to the mental lexicon, has been found in many studies to present an insignificant amplitude difference in dyslexics, demonstrating difficulties during these processing steps in dyslexia.

Lastly, the LPC component is associated with word recognition memory in tasks involving “old/new” word recognition. According to some authors, this component is also associated with access to the phonological representations of forms that do not have an entry in the orthographic lexicon (pseudowords, pseudohomophones). The reduced LPC amplitudes found in dyslexics may indicate difficulties in word recognition memory and/or impaired access to phonological representations.

Conversely, there are studies that did not find any differences in the abovementioned ERP amplitudes between dyslexic and non-dyslexic participants. Possible explanations for this inconsistency relate to subject screening, task differences, language transparency, and the presence of diverse reading impairment profiles in the dyslexic population.

In the second part of the review, we focused on investigations of eye movement patterns in dyslexic individuals. A large amount of data in studies of eye movements has shown that dyslexic individuals have abnormal eye movement patterns. Almost all sorts of eye movements (saccades, fixation, vergence, smooth pursuit (not described here) have been researched over the years, with dyslexic and non-dyslexic subjects showing different patterns. As far as saccades are concerned, many studies found that, during reading, many dyslexics presented more frequent saccades of smaller amplitude, as well as a high number of backward saccades. Apart from saccades, fixation has also been described as abnormal in dyslexic and non-dyslexic individuals. Dyslexics demonstrate a high number of longer-duration fixations and quite often present instability during fixation. Lastly, many studies found poor binocular coordination in dyslexics during and after the saccades in reading and non-reading tasks and during prolonged fixations. In his studies, Pavlidis assumed that the atypical eye movements found in dyslexic children during a non-reading task suggested a central and/or oculomotor impairment in dyslexia. However, other studies failed to replicate these results. This inconsistency in results may be due to subject selection criteria, the experimental procedure and/or the data analysis [60,81].

Some studies demonstrated that atypical eye movements were task dependent. Specifically, they found atypical eye movement patterns only during reading but not during non-reading tasks, and they surmised that, since ocular motor deficits are only related to reading and to higher-level processing, there is a deficiency at a higher psycholinguistic level of processing. This demonstration is in accordance with the phonological deficit theory. However, other studies do not replicate these findings since they found atypical eye movements in both reading and non-reading tasks.

There is no consistent evidence that can allow us to conclude on a standard processing profile of dyslexics, since inconsistent results are found in both electroencephalographic and eye movement registration studies. In this final part, we review factors that may contribute to this inconsistency in results, namely task differences, language transparency, subject screening and/or the presence of diverse reading impairment profiles in the dyslexic population.

First, the nature and difficulty of the task play a vital role in highlighting the impairments of dyslexic participants and can lead to different results concerning ERP amplitudes and latencies. Different tasks and different linguistic material activate different processing mechanisms and reveal different deficits found in dyslexia. As a result, some studies may find differences in ERP components between dyslexics and non-dyslexics, whereas others do not. In addition, smaller group differences have been reported in functional neuroimaging studies for easier phonological tasks compared to harder phonological tasks [82], implying that task difficulty is an essential component in identifying abnormal activation patterns [18].

Secondly, the differences may be related to orthographic transparency. According to Paulesu et al. [83], the severity of dyslexia can vary across different languages depending on the degree of orthographic transparency. Their study showed that the performance of Italian dyslexics (a transparent language) was better in reading tasks than the performance of French and English (non-transparent languages) dyslexic individuals. However, when compared to control participants from their countries, the three dyslexic groups were similarly impaired on reading and phonological tasks. Indeed, among the ERP studies presented in our review, differences in the ERP components have been mentioned independently of language transparency: reduced ERP amplitudes in dyslexics have been reported in both more transparent (e.g., Portuguese: [18,23], German: [17,19]) and less transparent languages (e.g., French: [20]).

Thirdly, another factor that may contribute to inconsistency in the results is the different and complex behavioral profiles observed. Dyslexics taking part in studies differed both with respect to age (kindergarten children with a high risk of dyslexia, children of different ages, preadolescents, adults) and to level of reading impairment. Some studies examined dyslexic participants who presented more severe deficits (over 2 SD below the mean reading time of normal readers) whereas others examined participants with milder deficits (1.29 SD below the mean reading time of normal readers). It should also be noted that many studies found different profiles within the same subgroup of dyslexics [21].

Furthermore, we cannot be sure if dyslexic participants have a mixed dyslexia deficit, involving both phonological and visuo-attentional deficits. As already mentioned in the introduction, each study using one of these two scientific techniques (ERP vs. EM) focuses a priori on a certain hypothesis about dyslexia (ERP for the phonological impairment hypothesis and eye movement recordings for the visuo-attentional deficit hypothesis). However, even if the two hypotheses may not be mutually exclusive, there is not sufficient evidence to prove them since these two hypotheses have not yet been examined simultaneously by using ERPs and eye tracking. Finally, in order to reach conclusions about the processing profile of dyslexic subjects, we need equally measurable methodological data, i.e., the same experimental task and material must be examined with the simultaneous use of the two abovementioned techniques. An in-depth screening process examining both phonological and visuo-attentional capacities is a prerequisite.

In conclusion, the importance of using the two techniques in order to examine the causes of dyslexia is manifold. Firstly, by combining ERP and eye movement recordings, we can better understand which processing steps during reading are disturbed in dyslexia (from a visual and oculomotor step to a higher linguistic processing step, i.e., the retrieval of linguistic information). Secondly, it can help to disentangle different profiles in the dyslexic population in terms of the weight of visuo-attentional and/or linguistic factors implicated in dyslexia. Thirdly, it allows to better understand dyslexia dysfunction to develop specific types of remediation for dyslexic subjects.

Several studies made by the group of Bucci [65,68,69,70] showed an abnormal oculomotor pattern in dyslexics, which could be due to visuo-attentional deficits already reported in dyslexia. For the first time, our aim is to show the importance of studying oculomotor and phonological capabilities in dyslexics at the same time, in order to explore more precisely where the deficit occurs. In other words, we make the hypothesis that we will find longer fixations and several saccades during reading words, and different types of non-words (pseudohomophones, pseudowords, consonant strings and symbol strings) but also abnormal ERP responses associated with different processes during reading (print processing by comparing alphabetic to non-alphabetic stimuli; phonological processing by comparing phonologically legal to phonologically illegal stimuli; lexical access by comparing stimuli that have an access to the mental lexicon to stimuli that do not; access to the phonological representations and word recognition memory by comparing stimuli that have an access to the phonological lexicon with stimuli that do not). We also predict to find, depending to the type of dyslexia (visuo-attentional or phonological or both), more pronounced oculomotor or phonological deficit, or both. Finally, these deficits may be attenuated for dyslexics who underwent re-education.

To our knowledge, no study has yet examined the two main hypotheses of dyslexia with the simultaneous use of both electroencephalography and eye movement recording techniques; this remains as an interesting challenge for future research.

## Figures and Tables

**Table 1:** Summary of the deficits in ERP components reported in dyslexic versus non-dyslexic participants.

ERP Components	Deficits Reported in Dyslexic Subjects with Respect to ERP Components	Level of Linguistic Analysis Studied	Studies That Found Differences in Dyslexic Subjects with Respect to ERP Components	Studies That Did Not Find Differences in Dyslexic Subjects with Respect to ERP Components
N170	Reduced level of print sensitivity and deficits in visual orthographic processing steps during reading	Lexicon	Maurer et al. [17], Araújo et al. [18], Hasko et al. [19], Mahé et al. [20], Shany and Breznitz [21],	Maurer et al. [22], Araújo et al. [23]
N320	Phonological processing deficit	Lexicon	Araújo et al. [23] Mahé et al. [24]	Araújo et al. [18]
N400	Deficient mechanism of grapheme–phoneme conversion Impaired access to the lexicon	Lexicon	Hasko et al. [19], Rüsseler et al. [25], Johannes et al. [26], McPherson et al. [27]	Bonte and Blomert [28], Silva-Pereyra et al. [29]
LPC	Impaired access to the phonological representations of words/difficulties in word recognition memory/unspecified phonological representations	Lexicon	Hasko et al. [19], Schulte-Körne et al. [30], Wachinger et al. [31]	

**Table 2:** Summary of the studies that found atypical and typical eye movement parameters in dyslexics.

Eye Movement Parameter in Dyslexics	Dyslexics > Non-Dyslexics		Dyslexics = Non-Dyslexics	
	Study	Type of the task	Study	Type of the task
		RT: reading task;		RT: reading task;
		NRT: Non-reading task;		NRT: Non-reading task;
		BOTH: both reading and non-reading task		BOTH: both reading and non-reading task
Number and amplitude of prosaccades	De Luca et al. [46]	BOTH (atypical EM only during the reading task)	Brown [47]	NRT
	Eden et al. [48]	NRT	Olson et al. [49]	BOTH
	Martos and Vila [50]	BOTH (atypical EM associated with text difficulty)	Black et al. [51]	NRT
	Fischer et al. [52]	NRT	Pirozzolo and Rayner [53]	RT (normal EM when given a text appropriate to their reading level)
	Rayner [54]	RT		
	Biscaldi et al. [55]	NRT		
	Biscaldi et al. [56]	NRT		
Number and amplitude of backward saccades	Martos and Vila [50]	BOTH (atypical EM associated with text difficulty)	Olson et al. [49]	BOTH
	Zangwill and Blakemore [57]	RT	Black et al. [51]	NRT
	Pavlidis [58]	NRT	Pirozzolo and Rayner [53]	RT (normal EM when given a text appropriate to their reading level)
	Pavlidis [59]	NRT	Stanley et al. [60]	NRT
Number and duration of fixations	De Luca et al. [46]	BOTH (atypical EM only during the reading task)	Olson et al. [49]	BOTH (similar EM only during the non-reading task)
	Eden et al. [48]	NRT	Hyona and Olson [61]	RT
	Martos and Vila [50]	BOTH (atypical EM associated with text difficulty)	Pirozzolo and Rayner [53]	RT (normal EM when given a text appropriate to their reading level)
	Rayner [54]	RT		
	Hutzler and Wimmer [62]	RT		
	Li et al. [63]	NRT		
	Adler-Grinberg and Stark [64]	BOTH		
	Bucci [65]	RT		
	Hatzidaki et al. [66]	BOTH (atypical EM only during the reading task)		
Poor binocular coordination	Bucci [65]	RT		
	Jainta and Kapoula [67]	RT		
	Tiadi et al. [68]	NRT		
	Bucci et al. [69]	BOTH (atypical eye movements during both tasks)		
	Bucci et al. [70]	BOTH (atypical eye movements during both tasks)		
	Kirkby et al. [71]	BOTH (atypical eye movements only during the reading task)		
	Trauzettel-Klosinski et al. [72]	BOTH (atypical eye movements only during the reading task)		

**Table 3:** Summary of studies that used both reading and non-reading tasks in order to study eye movement patterns of dyslexics.

Atypical Eye Movement Pattern in Both Reading and Non-Reading Tasks	Atypical Eye Movement Pattern Only in the Reading Task
Jainta and Kapoula [67]	Kirkby et al. [71]
Bucci et al. [69]	Trauzettel-Klosinski et al. [72]
Bucci et al. [70]	De Luca et al. [46]
	Hatzidaki et al. [66]
